# Auditing frontier general-purpose large language models in biomedical tasks: reasoning gains, extraction limits, and benchmark reliability

**DOI:** 10.21203/rs.3.rs-8605899/v1

**Published:** 2026-02-18

**Authors:** Yu Hou, Zaifu Zhan, Min Zeng, Yifan Wu, Shuang Zhou, Xiaoyi Chen, Huixue Zhou, Meijia Song, Rui Zhang

**Affiliations:** 1 Division of Computational Health Sciences, University of Minnesota, Minneapolis, Minnesota, USA.; 2 Department of Electrical and Computer Engineering, University of Minnesota, Minneapolis, MN, USA.; 3 School of Nursing, University of Minnesota, Minneapolis, MN, USA

## Abstract

As large language models approach clinical deployment, their deployment-relevant reliability and the validity of the benchmarks used to assess it remain insufficiently examined. Here, we present a unified, reproducible, and human-centric audit of frontier general-purpose language models using representative biomedical text-mining tasks and nine biomedical question-answering benchmarks spanning reasoning-intensive, extraction-oriented, and multimodal settings. We observe consistent gains in clinical reasoning and multimodal biomedical QA; however, limitations in format-constrained tasks such as span-level extraction and evidence-dense summarization pose challenges for integration into structured clinical workflows, despite narrowing gaps with supervised systems. Blinded expert adjudication confirms more coherent and clinically plausible reasoning and further reveals that a substantial fraction of apparent errors arises from outdated or ambiguous benchmark annotations, suggesting that current benchmarks may misestimate model capability and potentially misguide deployment decisions. Cost-normalized analyses demonstrate that recent frontier models achieve higher accuracy at substantially lower cost per correct answer, reshaping practical deployment trade-offs for scalable digital medicine systems. Together, these findings suggest that general-purpose language models are approaching deployment-relevant reliability; however, safe and effective clinical use will require hybrid architectures, external grounding, and human-in-the-loop evaluation and expert oversight.

## Introduction

The exponential growth of biomedical literature presents a fundamental challenge to scientific discovery and clinical practice. With PubMed indexing over 38 million records and adding more than one million articles annually, the capacity for human experts to curate knowledge and stay abreast of new findings has been far exceeded^1–3^. This information overload was starkly illustrated during the COVID-19 pandemic, which generated over 390,000 articles by the end of 2024 alone^4,5^. Consequently, the development of robust, automated biomedical natural language processing (BioNLP) tools has shifted from a convenience to a necessity^6–8^. While conventional supervised models have served as the bedrock of BioNLP, they are constrained by their reliance on large-scale manual annotation and their inability to generalize beyond narrow training distributions^9–12^. The emergence of large language models (LLMs), particularly transformer-based architectures with hundreds of billions of parameters, represents a paradigm shift in how we synthesize and reason with medical information^13^. Models such as GPT-3.5 and GPT-4 have already demonstrated remarkable linguistic comprehension and performance on medical licensing examinations^14,15^.

However, translating these general-purpose capabilities into reliable biomedical research and clinical tools remains a complex and unresolved endeavor. In real-world clinical settings, this gap directly affects trust, interpretability, and the risk of over-reliance on automated decision support. As model reasoning improves, it also becomes increasingly unclear whether observed errors arise from model failure or from limitations of static biomedical benchmarks themselves. Initial systematic evaluations have highlighted significant disparities in performance, with direct implications for clinical validation and deployment risk. For instance, Chen et al. demonstrated that while GPT-4 approaches state-of-the-art performance in question answering, it significantly lags behind task-specific fine-tuned models in precision-demanding tasks such as information extraction^16^. Similarly, evaluations of open-source models like DeepSeek reveal persistent trade-offs between precision and recall in event extraction^17^. Furthermore, the field faces a “moving target” problem: the rapid cadence of model updates, such as the release of the multimodal GPT-4 Omni (GPT-4o)^18^ and the reasoning-enhanced GPT-5 in August 2025—threatens to render static benchmarks obsolete. Crucially, as frontier models approach expert-level reasoning, static benchmarks increasingly impose a validity ceiling, conflating dataset noise with true model error.

Here, we present a comprehensive, longitudinal evaluation of frontier models—GPT-5 and GPT-4o—benchmarked against their predecessors and specialized systems. We extend the standardized evaluation protocol established by Chen et al.^16^ to cover a spectrum of five core BioNLP tasks (including named entity recognition and relation extraction) and expand the scope to include nine diverse question-answering (QA) datasets that test knowledge retrieval, clinical reasoning, and multimodal (visual) understanding^19–21^. This study goes beyond simple performance metrics to address four critical dimensions of clinical deployment. First, we quantify the “reasoning gap” between GPT-5 and GPT-4o in complex, multimodal biomedical scenarios, including tasks that require jointly interpreting radiology images and accompanying clinical text. Second, we assess the convergence of general-purpose LLMs toward the accuracy of domain-specific systems. Third, we analyze the practical trade-offs between inference cost, latency, and accuracy. Finally, and perhaps most importantly, we conduct a rigorous human audit of model-benchmark disagreements. Our findings not only map the performance trajectory of these frontier models but also expose the fragility of existing “gold standard” datasets, suggesting that future biomedical AI evaluation must evolve from static benchmarking to dynamic, human-AI collaborative auditing.

## Results

Performance diverged systematically across task regimes, revealing distinct strengths in reasoning-intensive settings and clinically relevant weaknesses in format-constrained tasks. The largest gains were observed in biomedical QA, particularly in reasoning-intensive and multimodal settings. In the five-shot setting, it improved mean accuracy by +0.111 over GPT-4o and showed more consistent performance across clinical domains and multimodal inputs. On the other hand, the five main BioNLP task families, including NER, RE, summarization, simplification, and document classification, saw only small improvements. GPT-5 made the gap smaller with fine-tuned task-specific baselines, but it didn’t completely close it, especially for tasks that focus on extraction and are sensitive to format. Human evaluation and cost-efficiency analyses provide additional context for these trends, revealing that GPT-5 offers more coherent explanations and a lower cost-per-correct-answer, even though it is still prone to grounding and format consistency failures.

### Heterogeneous performance in core NLP tasks reveals trade-offs in model behavior

**Figure 1:**
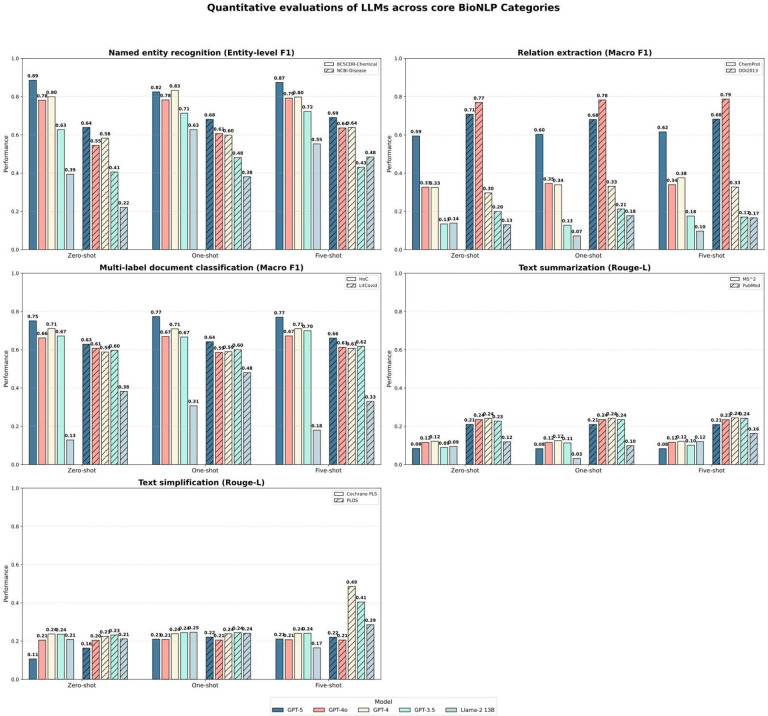
Evaluation of frontier and prior-generation language models across core BioNLP task families. Averaged performance across five task categories (Named Entity Recognition, Relation Extraction, Multi-label Document Classification, Summarization, and Text Simplification) under zero-shot, one-shot, and five-shot prompting. Bars show mean performance across datasets within each task category.

While frontier models continue to advance, our updated benchmark reveals a complex performance landscape across the five core BioNLP task families (Figure. 1). Unlike the uniform gains observed in reasoning tasks, GPT-5 did not consistently outperform its predecessors in traditional information extraction and generation tasks, highlighting a divergence between semantic reasoning capabilities and strict output formatting constraints.

Frontier models narrowed, but did not close, the gap with supervised systems in extraction tasks, underscoring persistent boundary-precision limitations. On BC5CDR-Chemical, performance approached the task-specific SOTA (five-shot F1: 0.874 vs. 0.950), yet accuracy on NCBI-Disease remained substantially lower (0.691 vs. 0.909), reflecting sensitivity to domain terminology. A similar pattern appeared in Relation Extraction (RE): GPT-5 improved results on ChemProt but was slightly outperformed by GPT-4o on DDI2013 (0.787 vs. 0.786, five-shot), indicating that schema alignment may influence model behavior as much as model scale.

Style-controlled generation tasks exposed the clearest divergence. On PLOS simplification, GPT-4 adapted effectively to example style (0.486), whereas GPT-5 performance fell markedly (0.221). Token-level analysis (Figure 6) suggests a verbosity-driven generation bias, producing detailed explanatory text even when brevity is required, beneficial in complex reasoning but detrimental in style-constrained applications. Summarization performance followed the same pattern: all general-purpose models generated high-level conceptual abstracts rather than evidence-dense summaries, yielding ROUGE-L scores near 0.21, far below fine-tuned SOTA baselines (0.43).

### Frontier models show consistent gains in clinical reasoning and multimodal biomedical QA

**Figure 2:**
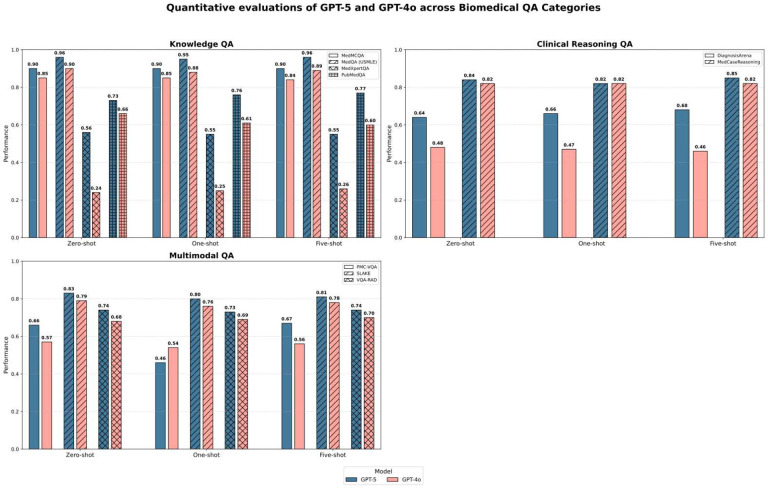
Accuracy of GPT-5 and GPT-4o across nine biomedical QA benchmarks. Averaged accuracy across knowledge-oriented, clinical-reasoning, and multimodal biomedical QA datasets. Bars show mean performance across datasets within each task category.

In contrast to the mixed performance observed in formatting-constrained BioNLP tasks, GPT-5 demonstrated a clear and consistent advantage in biomedical question answering (QA). Across nine diverse QA benchmarks (Figure 2), GPT-5 achieved substantial improvements over GPT-4o, particularly in the five-shot condition that best approximates real-world decision support (accuracy 0.768 vs. 0.657, Δ = +0.111). This advantage extended across cognitive domains rather than being confined to isolated task types. On knowledge-focused tasks such as MedQA (USMLE), GPT-5 reached near-ceiling performance (0.956). The performance gap widened further in reasoning-intensive tasks. On MedXpertQA and DiagnosisArena, GPT-5 exceeded GPT-4o by +0.288 and +0.221, respectively representing the largest performance differences observed across the benchmark. Improvements also extended to multimodal contexts: on the SLAKE visual QA dataset, GPT-5 achieved 0.808 accuracy, compared with 0.776 for GPT-4o, indicating enhanced alignment between visual cues and clinical semantics.

### Fine-grained analysis reveals gains driven by multi-step inference and spatial reasoning

**Figure 3:**
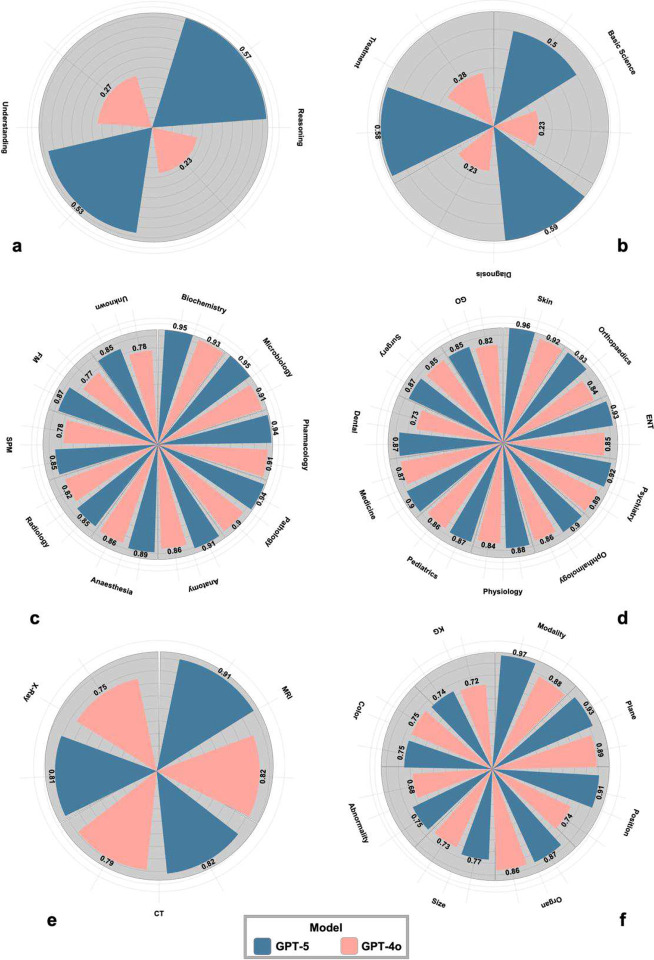
Fine-grained analysis of reasoning gains across biomedical QA subtypes. (a). Subtype-level comparison of GPT-5 versus GPT-4o across MedXpertQA question types (reasoning and understanding); (b). Subtype-level comparison of GPT-5 versus GPT-4o across MedXpertQA medical tasks (basic science, diagnosis, and treatment); (c). Subtype-level comparison of GPT-5 versus GPT-4o across MedMCQA clinical specialty; (d). Subtype-level comparison of GPT-5 versus GPT-4o across MedMCQA foundation medicine; (e). Subtype-level comparison of GPT-5 versus GPT-4o across SLAKE modality type; (f). Subtype-level comparison of GPT-5 versus GPT-4o across SLAKE content type.

Subtype analyses indicate that observed gains are primarily driven by multi-step inference and cross-modal semantic integration, rather than surface-level recall. A deeper breakdown of question subtypes explains where these improvements originate (Figure 3). Within MedXpertQA, the largest increases were observed in the Diagnosis (+0.36), Reasoning (+0.34), and Treatment (+0.30) categories, tasks that require multi-step inference and the synthesis of distributed biomedical evidence. Moderate, consistent gains were also observed in Basic Science (+0.27) and Understanding (+0.26) categories, suggesting improvements in foundational biomedical representation rather than isolated task adaptation.

The discipline-level decomposition of MedMCQA further reinforces this trend: GPT-5 outperformed GPT-4o across nearly all medical specialties, with the most pronounced improvements in Orthopedics (+0.09), Otolaryngology (+0.08), and Anatomy (+0.06), followed by steady gains in Ophthalmology (+0.05) and core preclinical domains such as Pharmacology, Microbiology, and Pathology (+0.03 to +0.04). This broad distribution suggests that gains arise from improved generalization rather than memorization of narrow domains.

Multimodal evaluation revealed similar patterns. In SLAKE, where visual reasoning intersects with textual interpretation, GPT-5 showed the largest benefits in categories requiring spatial judgment—Position (+0.17), Modality (+0.09), and Abnormality (+0.07)—whereas improvements were limited in surface-feature categories such as Organ and Color. Performance also varied by imaging modality, with the strongest improvement on MRI (0.909 vs. 0.818), followed by X-ray (Δ +0.06) and CT (Δ +0.02). Together, these analyses suggest that the key driver of GPT-5’s advantage is not rote recall, but improved reasoning, representation consistency, and cross-modal semantic integration.

### Economic viability and latency enable scalable deployment of frontier biomedical LLMs

**Figure 4:**
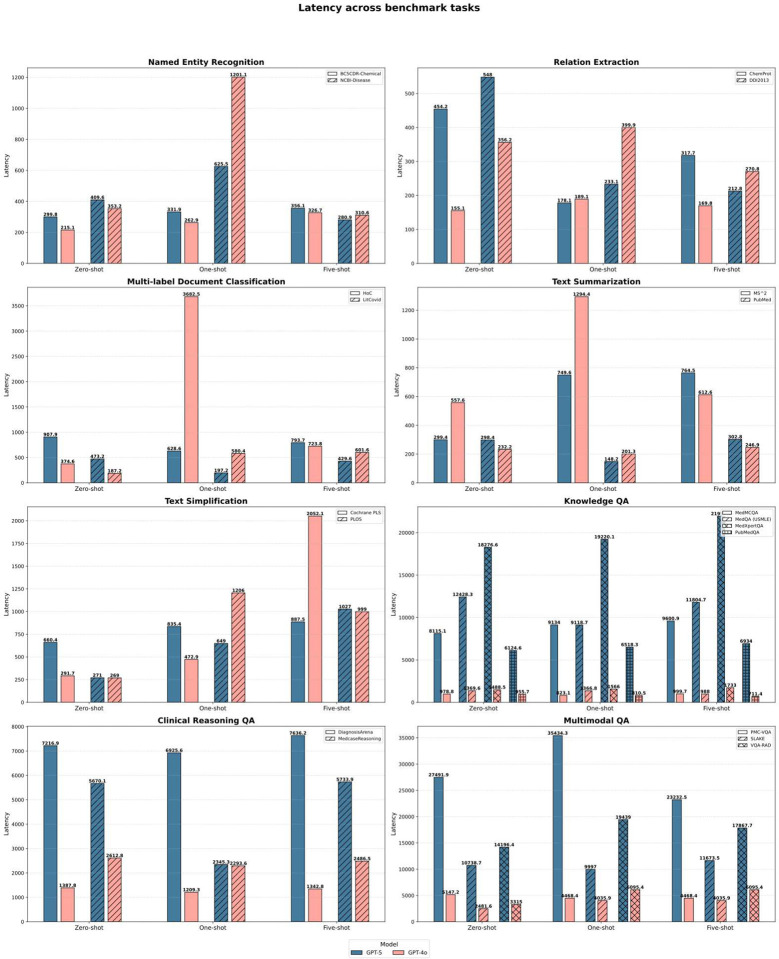
Latency across benchmark tasks for GPT-5 and GPT-4o under zero-shot, one-shot, and five-shot prompting.

**Figure 5:**
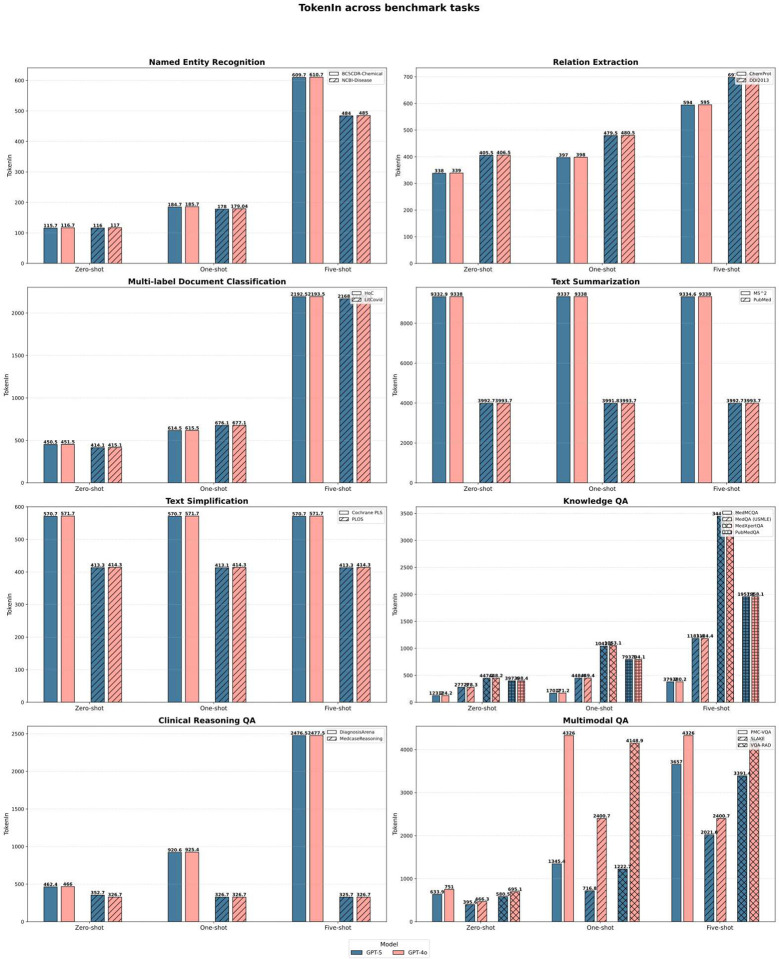
Token-in usage across benchmark tasks for GPT-5 and GPT-4o across prompting conditions.

**Figure 6:**
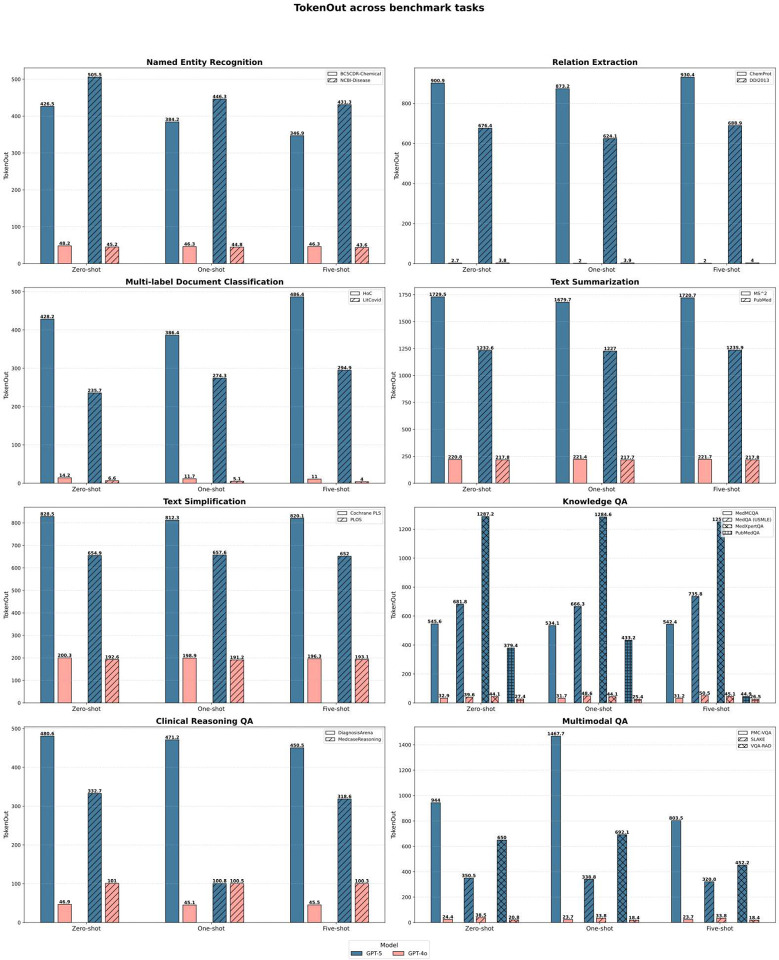
Token-out generation across benchmark tasks for GPT-5 and GPT-4o across prompting conditions.

To assess deployment relevance beyond accuracy, we evaluated cost, latency, and cost-normalized performance across representative biomedical workloads. Benchmarking cost and efficiency revealed that GPT-5 offers not only higher accuracy but also improved deployment economics, particularly in high-throughput biomedical use cases (Figure 4–6). To align the evaluation with realistic operational constraints, we implemented two deployment pathways reflecting how large models are commonly integrated into biomedical systems. First, the five core BioNLP tasks were executed using the batch API, a mode optimized for large-scale text processing pipelines such as entity mining, automated annotation, and retrospective literature screening. Batch API pricing is substantially lower and can amortize token usage across thousands of requests, making it the economically dominant strategy for workloads in research networks, data warehouses, and population-level analytics. Second, biomedical QA tasks were evaluated using the standard API to emulate interactive clinical or decision-support scenarios, where responsiveness rather than throughput is the primary constraint. This separation enabled a more realistic comparison of model behavior under conditions that approximate typical deployment environments—high-volume back-end processing versus low-latency, user-facing inference.

On core text mining workloads, GPT-5 produced longer and more detailed outputs than GPT-4o while maintaining similar input token demands. Despite increased output length, batch pricing resulted in a 45–50% reduction in total processing cost. For example, processing 1,000 instances of BC5CDR-Chemical or DDI2013 decreased from approximately $0.0036 to $0.0019. Latency remained stable at 300–450 ms, indicating that the additional content did not impose measurable runtime penalties.

Cost differences were more pronounced in biomedical QA scenarios. Under standard API pricing, GPT-5 required fewer tokens and retained a lower unit price ($1.25 input / $10 output) than GPT-4o ($2.50 / $10). After normalizing for token volume and accuracy, processing 100 QA samples averaged $0.72 for GPT-5 versus $1.06 for GPT-4o, corresponding to an estimated 30–40% reduction in cost per correct answer. Latency remained comparable (typically 300–500 ms), suggesting that the model’s improved reasoning did not incur efficiency trade-offs. Together, these results indicate that frontier models can deliver both higher accuracy and improved cost efficiency, reshaping deployment trade-offs in biomedical AI systems, particularly those involving large-scale inference or cost-constrained clinical environments.

### Human evaluation confirms superior reasoning quality and reveals benchmark-dependent error signatures

Blind expert adjudication corroborated the automated performance gap between models. Inter-rater reliability was high across all scoring dimensions (logic-coherence ICC = 0.83–0.94; error-type κ = 0.82–0.91), and agreement on comparative correctness approached perfection (κ = 0.81–0.93), confirming score stability independent of evaluator. A two-way ANOVA demonstrated a significant main effect of model on explanation quality (p < 0.001): GPT-5 responses were consistently rated as more coherent and clinically grounded (mean 3.38 ± 0.87) compared to GPT-4o (2.93 ± 0.85). Although rater-level variance was detectable (p < 0.001), the absence of a model × rater interaction (p = 0.978) indicates that all evaluators independently preferred GPT-5 outputs. Error distributions further diverged between systems. A χ^2^ analysis confirmed significant differences in failure modes (χ^2^ (4) = 289.90, p < 0.001), with reasoning errors dominating for both models (GPT-5: 62.4%; GPT-4o: 59.9%). Collectively, these results indicate that GPT-5 generates explanations that are more logically structured, medically consistent, and less prone to semantic misunderstanding than GPT-4o.

### Clinical risk of flawed benchmarks: structural limitations in static biomedical evaluation

Inspection of disagreement cases, instances where GPT-5 was mismarked despite clinically plausible reasoning, exposed gold-label limitations and benchmark drift within existing evaluation frameworks. Four recurrent sources of mismatch emerged: (i) outdated clinical standards, (ii) ambiguous or multi-valid label sets, (iii) oversimplified answer formats, and (iv) annotation errors. These cases demonstrate that the benchmark’s “gold labels” do not always reflect contemporary medical consensus or diagnostic nuance, suggesting that benchmark maintenance, rather than model scaling alone, is becoming a critical bottleneck that complicates clinical validation and obscures meaningful performance gains as model reasoning improves. Expert review confirmed that, in multiple instances, GPT-5 generated responses that were preferable to the reference answer from a clinical standpoint, suggesting that the model’s performance may already exceed the reliability bounds of some static datasets. Together, these findings highlight a critical limitation: as LLM reasoning improves, static benchmarks impose a validity ceiling, conflating dataset noise with model error and obscuring meaningful performance gains.

## Discussion

Frontier biomedical LLM performance is no longer defined solely by accuracy benchmarks but by questions of reliability, deployment context, and systems integration. In this study, we evaluated GPT-5 as a general-purpose biomedical language model using a unified framework spanning five core BioNLP tasks and nine biomedical QA benchmarks. GPT-5 demonstrated substantial improvements in clinical reasoning, multimodal QA, and knowledge-based tasks, while still trailing domain-specialized systems on extractive and evidence-dense workloads. These findings suggest a transition point in the field: general-purpose models now perform well enough that the central question is shifting from whether they are competitive to where, how, and under what constraints they can be responsibly deployed. Rather than replacing specialized systems outright, frontier language models are best positioned as flexible reasoning layers within hybrid clinical and biomedical information systems. They are useful when interpretability, adaptability, and cross-task generalization matter, but still require safeguards and augmentation for high-precision, format-constrained tasks.

Emerging evidence from recent evaluations supports our findings and aligns with a growing body of literature indicating that, when carefully prompted, general-purpose LLMs can approach or even exceed domain-specific systems in medical reasoning and explanatory writing tasks, such as MedQA and plain-language summarization^22,23^. A particularly relevant comparison is a recent assessment of GPT-5 on the MedXpertQA-Text benchmark^24^, which reported similar accuracy levels despite using explicit chain-of-thought (CoT) prompting, whereas our evaluation relied solely on simple, single-turn prompts. This convergence suggests that the baseline reasoning capacity of GPT-5 approaches levels previously accessible only through structured CoT scaffolding. From an applied standpoint, this shift has practical consequences. For many structured biomedical QA tasks, direct prompting may now be sufficient to achieve competitive performance, especially in settings where computational efficiency, throughput, and scalability matter. However, modest yet consistent improvements observed under CoT prompting in prior work indicate that structured reasoning templates may still be beneficial for high-stakes or diagnostically complex tasks requiring explicit justification. Together, these findings support a tiered prompting paradigm, where direct prompting serves as the default for large-scale or cost-sensitive workflows, with CoT-guided prompting reserved for contexts that demand reasoning transparency or interpretability. Despite these gains, certain task families remain resistant to prompt-based improvement. Consistent with previous reports^25–27^, summarization and span-level extraction tasks continue to reveal persistent weaknesses, including incomplete content coverage, imprecise boundary control, and instability in output length. These tasks rely less on conceptual reasoning and more on fine-grained linguistic fidelity—an area where general-purpose LLMs still lag domain-adapted or supervised architectures. In such cases, hybrid approaches integrating retrieval, domain ontologies, or structured supervision remain necessary to achieve the accuracy and consistency required for clinical-grade biomedical text processing.

**Figure 7:**
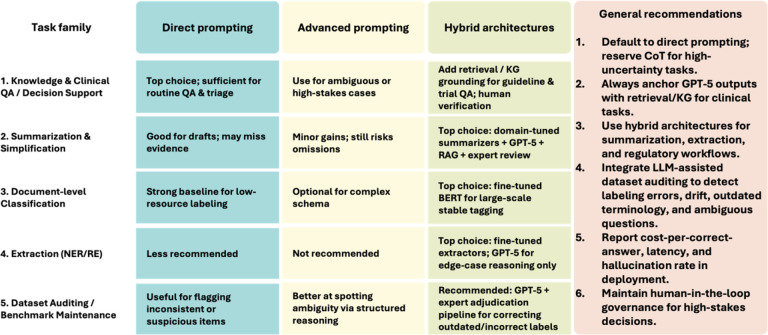
Recommended deployment patterns for GPT-5 across biomedical NLP applications. (i) Direct prompting, where the model is asked to produce an answer without structured reasoning scaffolds; (ii)Advanced prompting, including CoT, schema-guided reasoning, and exemplar-based prompting; and (iii) Hybrid architectures, where GPT-5 is paired with domain-tuned extractors, retrieval-augmented systems, or expert oversight.

At the deployment layer, our findings support a differentiated strategy for model integration that aligns prompting complexity with task demands (Figure 7). Biomedical question answering and guideline-based decision support benefit most from direct prompting, with optional escalation to advanced prompting (CoT or exemplar-guided reasoning) in cases that are ambiguous or high-stakes. Further grounding with retrieval from institutional guidelines, formularies, or biomedical knowledge graphs enhances traceability and reduces the risk of hallucination in regulated settings. Summarization and simplification tasks show limited benefit from advanced prompting alone. Instead, hybrid architectures—such as retrieval-augmented generation combined with domain-tuned summarizers and expert verification—remain the most reliable approach, particularly for evidence-dense or trial-level content. Document-level classification can be effectively supported with direct or few-shot prompting in rapidly evolving or low-resource label environments. However, when label schemas stabilize and throughput requirements increase, fine-tuned BERT-derived models continue to outperform general-purpose LLMs in cost-efficiency and consistency, making them preferable for production workflows. Span-level extraction tasks (NER and RE) remain the most challenging category for general-purpose LLMs. Advanced prompting provides only a marginal benefit, and boundary precision failures persist. As a result, domain-tuned extractors remain the primary engines for large-scale extraction, with GPT-5 best positioned as an adjudication layer for schema drift, ambiguous instances, or suspected annotation errors.

A deeper comparison with prior work clarifies where our findings reinforce existing trends and where they diverge. First, consistent with earlier observations from GPT-4 series and clinically tuned models, we confirm that scaling and alignment continue to improve medical QA and explanatory reasoning performance^22,23,28–30^. Nonetheless, our findings indicate that the incremental enhancements noted at GPT-4 are more pronounced at GPT-5, with advancements spanning reasoning categories and modalities rather than being confined to specific datasets. Second, our results are in line with reports that adding more examples doesn’t help as much as it used to as the model’s ability grows^22,31^. Few-shot prompting resulted in only marginal enhancements for GPT-5 and GPT-4o, primarily confined to tasks that are sensitive to stylistic control. On the other hand, reasoning-heavy and multimodal QA tasks did very well even with little prompting. This suggests that frontier models are relying more on intrinsic priors than on prompt-provided scaffolding. Third, our findings contrast with studies indicating negligible enhancements beyond GPT-4; various methodological distinctions may elucidate this divergence, including stricter prompt discipline, more granular task decomposition, and the implementation of cost-normalized metrics. The latter is particularly consequential: even when raw accuracies are similar, operational conclusions may differ once latency, pricing, and cost-per-correct-answer are accounted for. Finally, our economic analysis complements accuracy-centric reports by demonstrating that GPT-5 delivers 30–50% lower cost-per-correct answer relative to GPT-4o under matched evaluation conditions. This shift transforms the assessment from a performance comparison into a deployment calculus, one in which capability, cost, and latency jointly determine the suitability of the model.

Not all apparent model failures reflected genuine errors; many instead exposed weaknesses in the benchmarks themselves. Although GPT-5 was consistently rated as more coherent and clinically plausible, expert review revealed that a substantial portion of model–label mismatches arose from limitations in dataset design rather than reasoning faults. In MedCaseReasoning, GPT-5 frequently produced clinically preferred or more granular diagnoses compared with the gold labels. For example, Adult PFAPA syndrome versus the generic PFAPA syndrome, Spindle cell carcinoma of the breast versus “SpindleCellCarcinoma”, and Starvation-induced hepatocellular injury rather than Starvation-induced liver enzyme elevation. Similar issues appeared in PubMedQA, where nuanced or conditionally supported results, such as studies on sleep bruxism or population-specific mental-health findings were forced into rigid yes/no/maybe labels. Experts also identified outdated terminology, overlapping diagnostic categories, and occasional annotation errors, indicating that benchmark drift and label rigidity can mask model capability as systems advance. As frontier models become increasingly precise, the limitations of static bench-marks become more visible, shifting the locus of error from model to dataset. These findings suggest a forward path: using high-performing LLMs as auditing tools to identify ambiguous items, detect outdated terminology, and flag label inconsistency for targeted expert review. Rather than replacing human oversight, such workflows could support continuous benchmark maintenance, reduce annotation burden, and ensure that evaluation frameworks remain aligned with contemporary clinical language and reasoning standards. In this light, GPT-5 and similar models may function not only as reasoning engines but also as instruments for diagnostic dataset curation and evidence standardization.

From a systems perspective, hallucination, evidence inconsistency, and missing citations remain key obstacles to safe clinical deployment of high-capacity LLMs. These issues are not resolved by prompt engineering alone; rather, they reflect deeper gaps in grounding and provenance control documented across summarization, classification, and extraction tasks^25–27^. Our findings, together with evidence from retrieval-augmented and knowledge-graph-based approaches, support a hybrid architectural model in which GPT-5 functions as a controllable reasoning layer rather than as an unconstrained generator. In such configurations, authoritative external resources, such as retrieved literature, biomedical ontologies, or institutional knowledge graphs, provide structured grounding, while dedicated modules handle citation verification, document provenance, and conflict resolution^32–34^. This design pattern is particularly relevant for high-stakes applications, such as guideline QA, clinical trial eligibility matching, medication safety, and complex decision-support workflows, where traceability and auditability are operational requirements rather than optional safeguards. In these settings, accuracy alone is insufficient; clinical deployment requires outputs whose evidence path can be inspected, validated, and trusted.

Several limitations qualify the interpretation and generalizability of our findings. First, the present evaluation focused primarily on English-language, text-dominant tasks, with only limited assessment of radiology-style visual input. Real-world clinical systems must operate across multilingual documentation, diverse imaging modalities, and EHR-specific formatting variability. Second, because evaluations were conducted through proprietary inference APIs, we lacked visibility into training distributions, alignment data, and system-level overrides, which constrains interpretability and limits fine-grained safety analysis. Third, although our prompting protocol was standardized to ensure comparability, it did not explore the rapidly expanding design space of dynamic retrieval workflows, structured reasoning templates, tool-augmented prompts, or calibration mechanisms. Finally, although blinded domain-expert adjudication strengthened the qualitative evaluation, the number of audited samples remained modest relative to the breadth and heterogeneity of tasks, and our study did not assess downstream clinical consequences.

These limitations point to concrete next steps in both benchmarking methodology and deployment practice. Future evaluation frameworks should combine automatic metrics with structured expert review to assess factual faithfulness, clinically harmful error modes, and semantic adequacy, particularly for summarization and extraction tasks where surface correctness may obscure reasoning failures^25,26^. In settings requiring high boundary precision or evidence completeness, hybrid architectures that integrate GPT-5 with retrieval augmentation, ontology-aware constraint checking, or knowledge graph verification are likely to provide more reliable and auditable performance^32–34^. For QA and clinical decision support, deployment should adopt cost-aware configurations that leverage batch inference for high-throughput pipelines and standard endpoints for interactive workflows, evaluating systems on cost-per-correct-answer and latency rather than token volume alone. Finally, reporting conventions should include token-normalized costs, throughput characteristics, and timing variance to support realistic capacity planning, as modest differences in accuracy may be outweighed by substantial economic or operational efficiencies in practice.

Looking forward, three priorities appear most consequential for advancing the evaluation and deployment of biomedical LLMs. First, future assessments must move beyond static and English-only QA benchmarks toward settings that reflect real clinical complexity, including multilingual documentation, multimodal evidence, temporal patient data, and imaging-structured narratives. Second, our findings support the need for hybrid evaluation and deployment architectures that combine retrieval-augmented reasoning, external tool execution, and knowledge-graph grounding within a unified framework. Such designs can help distinguish reasoning failure from evidence insufficiency, reduce hallucination risk, and improve factual traceability, particularly in explanation-heavy tasks highlighted in our expert review. Third, translation to practice will require prospective, human-in-the-loop studies that pair cost-normalized performance metrics with domain-expert adjudication and clinically meaningful endpoints. The annotation errors, outdated terminology, and scheme rigidity uncovered in widely used benchmarks emphasize that performance gains alone do not equate to clinical readiness.

Taken together, our results suggest that frontier general-purpose language models represent a meaningful shift for digital medicine, not as standalone solutions, but as reasoning scaffolds within human-centered, retrieval-grounded, and auditable clinical workflows. With careful integration of ontology-based verification, structured retrieval, and expert review, such systems have the potential to deliver accurate, cost-efficient, and trustworthy biomedical applications at scale. Implications for evaluation and governance: our findings indicate that automated benchmark scores alone are insufficient to support high-stakes clinical adoption. We recommend hybrid evaluation pipelines that combine benchmark auditing, targeted expert adjudication, and external grounding to improve reliability, traceability, and trust in digital medicine applications.

## Materials & Methods

### Benchmark scope and task families

We evaluated GPT-5 and GPT-4o across five core biomedical NLP task families: named entity recognition (NER), relation extraction (RE), multi-label document classification, text summarization, and text simplification. Additionally, nine biomedical question-answering (QA) datasets, spanning knowledge-based reasoning, clinical decision-making, and multimodal visual interpretation, were included. All datasets used were publicly available. For QA tasks, answer format definitions followed the native benchmark conventions (multiple choice and binary yes/no/maybe). Visual QA datasets (VQA-RAD and SLAKE) were evaluated using paired image–question–answer triplets. No dataset was altered beyond formatting for standardized prompting. Task definitions, dataset metadata, and licensing status are summarized in Table 1.

### Models evaluated and experimental setup

This study systematically evaluated the performance of GPT-5 and GPT-4o across all benchmark tasks utilizing a standardized and replicable protocol. The official OpenAI APIs were used to make predictions directly, which ensured they were the same as those made in production-grade inference settings. There were two operational configurations for GPT-5 and GPT-4o. The standard endpoint was used for QA and reasoning-intensive tasks, while the batch API was applied to core BioNLP tasks to optimize cost and latency. This distinction aligns with practical deployment scenarios and provides a basis for analyzing the performance–efficiency trade-offs reported in the cost evaluation section. To ensure consistency and traceability, we systematically recorded token usage and inference cost for every model–task pair. For each generation, the total number of input and output tokens was logged through the API metadata, allowing precise calculation of token-normalized cost according to the official OpenAI pricing for both standard and batch endpoints. These statistics were aggregated at the dataset and task levels to quantify efficiency alongside accuracy. In addition, latency—defined as the total time between prompt submission and response completion was automatically measured for each batch.

As historical baselines, results for GPT-4, GPT-3.5 and state-of-the-art (SOTA) task-specific baselines were obtained from the prior benchmark study by Chen et al.^16^. Those evaluations utilized the same prompt templates, decoding parameters, and dataset partitions, which enabled direct model comparison without rerunning the old systems. This alignment enables meaningful longitudinal evaluations of model improvement across different generations of LLMs. It also ensures that any improvements are due to architectural and alignment changes, not changes in protocol.

We tested all the models in three standard ways: zero-shot, one-shot, and five-shot. In the zero-shot condition, models received only the task description and input text, testing their generalization without exposure to examples. In the one-shot and five-shot settings, prompts additionally included one or five representative input–output exemplars drawn from the training split of each dataset. The same exemplars were used across all models and kept fixed throughout the study to eliminate bias from exemplar selection. This design isolates performance differences attributable to model reasoning and alignment rather than prompt variability.

For selected QA datasets that contained explicit subtype annotations (e.g., clinical reasoning type, question modality, or content domain), we further performed subject-level error and accuracy analyses. Each question instance was categorized by its underlying subtype (e.g., anatomy, diagnosis, pharmacology, image-based reasoning), enabling fine-grained comparison of GPT-5 and GPT-4o across reasoning dimensions. These analyses offered further clarity regarding the areas of most significant model enhancement and the biomedical knowledge categories that continued to pose difficulties.

The experimental setup overall replicates and builds upon the protocol set up in the last benchmark, ensuring that task definitions, decoding parameters, and evaluation metrics are all consistent. This unified framework establishes a controlled environment for comparing large language models across various biomedical NLP tasks. It also systematically combines measurements of accuracy, latency, and cost so that cross-model evaluation is clear and repeatable.

### Human evaluation of model explanations

To quantitatively assess the interpretability and reliability of LLM reasoning, a human evaluation study was conducted using a blind, cross-model design. Four medical experts were divided into two groups, each independently evaluating a distinct subset of 99 mispredicted items sampled from nine biomedical QA datasets. Each item consisted of the original question, the gold-standard answer, and the generated answers from both models, accompanied by brief self-explanations. To ensure unbiased assessment, model identities (GPT-5 vs. GPT-4o) were randomly masked as Model A or Model B for each sample following standard blind review protocols.

Experts rated the logical coherence of each explanation on a 5-point Likert scale (1 = illogical, 5 = highly coherent) and categorized each model’s error type as Hallucination, Reasoning error, or Misunderstanding of the question, a typology commonly used in LLM error-diagnostic frameworks. Additionally, raters judged the comparative correctness between the two models on a 5-point scale (1 = Model A is much closer to correct, 3 = equal, 5 = Model B is much closer).

Each sample was independently scored by two experts to enable reliability estimation. Inter-rater reliability for continuous logic-coherence ratings was quantified using a two-way mixed-effects ICC(3, k) model^51^, while categorical agreement for error-type and comparative-correctness judgments was assessed via Cohen’s κ^52^. Descriptive statistics (mean ± SD) were computed for each metric. To examine model-level differences, a two-way ANOVA was applied to logic-score data. Finally, inter-model variation in error-type distributions was evaluated using a Chi-square test of independence.

## Figures and Tables

**Table 1.** Evaluation datasets and metrics.

Datasets	Description	Training	Validation	Testing	Metrics
**Named Entity Recognition**					
BC5CDR-chemical^35^	PubMed articles annotated with mentions of chemicals and drugs.	4,560	4,581	4,797	Entity-level F1
NCBI-disease^36^	PubMed abstracts annotated with disease mentions and mapped to standardized disease concepts.	5,424	923	940	Entity-level F1
**Relation Extraction**					
ChemProt^37^	PubMed abstracts annotated with chemical–protein interaction relations across multiple categories.	19,460	11,820	16,943	Macro-F1
DDI2013^38^	DrugBank and Medline texts annotated for drug–drug interaction types.	18,779	7,244	5,761	Macro-F1
**Multi-label Document Classification**					
HoC^39^	Biomedical abstracts annotated with one or more of ten cancer hallmark categories.	1,108	157	315	Macro-F1
LitCovid^40^	A curated literature hub to track up-to-date multi-label topic classification corpus of COVID-19 articles in PubMed.	24,960	6,239	2,500	Macro-F1
**Text Summarization**					
PubMed Summarization^41^	Full biomedical articles paired with their expert-written abstracts, supporting single-document summarization.	117,108	6,631	6,658	Rouge-L
MS^2^42^	Collections of primary research articles aligned with systematic review summaries, requiring synthesis across documents.	14,188	2,021	-	Rouge-L
**Text Simplification**					
Cochrane PLS^43^	Systematic review abstracts paired with corresponding plain-language summaries written for lay readers.	3,568	411	480	Rouge-L
PLOS Simplification^44^	Scientific abstracts from PLOS journals paired with author-provided lay summaries.	26,124	1,000	1,000	Rouge-L
**Knowledge QA**					
MedQA (USMLE)^45^	The canonical medical licensing exam QA is widely used for LLM medical knowledge and reasoning.	10,177	1,271	1,271	Accuracy
MedMCQA^19^	A large-scale Multiple-Choice QA dataset sourced from real-world medical entrance exams, designed to test LLM medical knowledge and reasoning.	182,822	6,150	4,183	Accuracy
PubMedQA^46^	Biomedical research questions paired with PubMed abstracts.	190,142	21,127	500	Accuracy
MedXpertQA^47^ (text)	A comprehensive benchmark featuring both text and multimodal subsets, designed to evaluate LLMs’ expert-level medical knowledge and advanced clinical reasoning.	-	5	2,450	Accuracy
**Clinical reasoning QA**					
DiagnosisArena^48^	A dataset derived from clinical case reports in top-tier medical journals is designed to rigorously assess LLMs’ professional-level diagnostic reasoning capabilities in complex clinical scenarios.	-	-	915	Accuracy
MedcaseReasoning^49^	The dataset containing diagnostic QA cases paired with detailed clinician-authored reasoning is designed to evaluate and improve LLMs’ ability to align with professional diagnostic reasoning processes.	13,092	500	897	Accuracy
**Multimodal QA**					
VQA-RAD^20^	A dataset of clinically generated visual QA about radiology images.	1,793	-	451	Accuracy
PMC-VQA^50^	A large-scale medical Visual QA dataset featuring QA pairs derived from images across various modalities and diseases from PubMed Central.	176,948	5,000	-	Accuracy
SLAKE^21^	A large dataset for medical visual QA, featuring comprehensive semantic labels annotated by experienced physicians and a new structural medical knowledge base.	9,849	2,109	2,070	Accuracy

## Data Availability

All datasets used in this study are publicly available benchmark resources. The named entity recognition, relation extraction, document classification, summarization, and text simplification datasets were obtained from their original public releases, including BC5CDR, NCBI-Disease, ChemProt, DDI2013, HoC, LitCovid, PubMed Summarization, MS^2, Cochrane PLS, and PLOS Simplification. Biomedical question-answering benchmarks, including MedQA (USMLE), MedMCQA, PubMedQA, MedXpertQA, DiagnosisArena, MedCaseReasoning, VQA-RAD, PMC-VQA, and SLAKE, were likewise accessed through their publicly available distributions. No modifications were made to the original datasets beyond formatting required for standardized prompting and evaluation. All datasets contain no identifiable patient information and are distributed under licenses permitting academic research use. Detailed dataset descriptions, citations, and access links are provided in the Methods and References sections.

## Code availability

The codes are publicly available via https://github.com/houyurain/Frontier-LLMs-evaluation.
